# Chronic Oral Anticoagulation and Clinical Outcome in Hospitalized COVID-19 Patients

**DOI:** 10.1007/s10557-021-07194-y

**Published:** 2021-05-14

**Authors:** Vincenzo Russo, Roberta Bottino, Antonello D’Andrea, Angelo Silverio, Marco Di Maio, Paolo Golino, Gerardo Nigro, Orazio Valsecchi, Emilio Attena, Mario Enrico Canonico, Gennaro Galasso, Guido Parodi, Fernando Scudiero

**Affiliations:** 1 Cardiology Unit, Department of Translational Medical Sciences, University of Campania “Luigi Vanvitelli” - Monaldi Hospital, Via. Bianchi, 80131 Naples, Italy; 2 Department of Cardiology, Umberto I Hospital, 84014 Nocera Inferiore, Italy; 3 Cardiovascolar and Thoracic Department, Division of Cardiology, San Giovanni di Dio e Ruggi d’Aragona University Hospital, Salerno, Italy; 4 Division of Cardiology, Maria SS. Addolorata Hospital, Eboli, Salerno Italy; 5 Division of Cardiology, “Bolognini” Hospital, ASST Bergamo Est, Seriate, BG Italy; 6 Department of Cardiology, Monaldi Hospital, Naples, Italy; 7 Clinical and Interventional Cardiology, Sassari University Hospital, Sassari, Italy

**Keywords:** COVID-19, SARS-CoV-2, NOACs, VKA, ARDS, Outcome

## Abstract

**Purpose:**

The clinical course of COVID-19 may be complicated by acute respiratory distress syndrome (ARDS) and thromboembolic events, which are associated with high risk of mortality. Although previous studies reported a lower rate of death in patients treated with heparin, the potential benefit of chronic oral anticoagulation therapy (OAT) remains unknown. We aimed to investigate the association between OAT with the risk of ARDS and mortality in hospitalized patients with COVID-19.

**Methods:**

This is a multicenter retrospective Italian study including consecutive patients hospitalized for COVID-19 from March 1 to April 22, 2020, at six Italian hospitals. Patients were divided into two groups according to the chronic assumption of oral anticoagulants.

**Results:**

Overall, 427 patients were included; 87 patients (19%) were in the OAT group. Of them, 54 patients (13%) were on treatment with non-vitamin k oral anticoagulants (NOACs) and 33 (8%) with vitamin-K antagonists (VKAs). OAT patients were older and had a higher rate of hypertension, diabetes, and coronary artery disease compared to No-OAT group. The rate of ARDS at admission (26% vs 28%, *P*=0.834), or developed during the hospitalization (9% vs 10%, *P*=0.915), was similar between study groups; in-hospital mortality (22% vs 26%, *P*=0.395) was also comparable. After balancing for potential confounders by using the propensity score matching technique, no differences were found in term of clinical outcome between OAT and No-OAT patients

**Conclusion:**

Oral anticoagulation therapy, either NOACs or VKAs, did not influence the risk of ARDS or death in patients hospitalized with COVID-19.

## Introduction

Severe acute respiratory syndrome coronavirus 2 (SARS-CoV-2) is a novel human coronavirus recently recognized as the cause of the coronavirus disease 2019 (COVID-19). Despite the fast-growing understanding on the clinical features and natural history of COVID-19 [1], its pathophysiology is still debated and treatment remains largely empirical or based on observational evidences [2–7]. Although poorly symptomatic in many cases, COVID-19 may be complicated by severe conditions such as adult respiratory distress syndrome (ARDS), sepsis, and death [8]. An increasing number of studies have showed abnormal serum coagulation parameters in hospitalized patients with severe forms of COVID-19 with a trend toward hypercoagulable state [9–12], and may justify the high prevalence of venous thromboembolism (VTE), disseminated intravascular coagulation (DIC), and ARDS [9, 13–15]. Since anticoagulation treatment has been associated with lower risk of mortality in patients with severe form of COVID-19 [16], pharmacological antithrombotic prophylaxis may be considered in hospitalized patients in the absence of contraindications [17–19]. However, the benefit of chronic oral anticoagulation therapy (OAT) on clinical outcome of hospitalized patients with COVID-19 is still debated [20, 21].

The objectives of this study were to describe the prevalence in the use of OAT in hospitalized COVID-19 patients and to assess the association between OAT and the risk of ARDS, either at admission or developed during hospitalization, and in-hospital mortality.

## Materials and Methods

We retrospectively evaluated a cohort of 467 patients with laboratory confirmed COVID-19 admitted from March 1 to April 22, 2020 at six Italian Hospitals (Monaldi Hospital of Naples, Fatebenefratelli Hospital of Naples, ASST Bergamo East Hospital, Umberto I Hospital of Nocera Inferiore, University Hospital of Salerno, Sassari University Hospital). COVID-19 diagnosis was initially based on the World Health Organization criteria and all cases were later confirmed by real time reverse transcriptase–polymerase chain reaction analysis of throat swab specimens [22].

The study population was divided according to the pre-admission OAT use into two groups. All OAT patients were on treatment for at least 4 weeks before being admitted to the hospital.

The discontinuation, switch, or initiation of OAT during the hospitalization was considered exclusion criteria.

Treatment with low molecular weight heparin (LMWH) at admission was also considered an exclusion criterion.

This study was conducted according to the Declaration of Helsinki and approved by the institutional ethics committees. The requirement for informed consent from individual patients was waived due to the observational retrospective design of this study.

In all patients, demographic (age, gender, height and weight), clinical (comorbidities, pharmacological therapy before and during hospitalization), in-hospital course (admission in intensive care unit and respiratory support measures), complications (ARDS at admission or developed during hospitalization), and mortality were prospectively collected and recorded on an electronic datasheet. ARDS diagnosis was defined according to the Berlin definition [23].

### Statistical Analysis

Distribution of continuous data was tested with the Kolmogorov–Smirnov and the Shapiro-Wilk test. Normally distributed variables were expressed as mean ± standard deviation (SD), whereas non-normal distributed ones as median and interquartile range (IQR). Categorical variables were reported as numbers and percentages. Continuous normally distributed variables were compared by using the Student t-test; differences between non-normally distributed variables were tested with the Mann-Whitney *U* test. Categorical variables were compared with chi-squared test, or Fisher exact test, as appropriate.

Preliminary matching procedures were performed to obtain a covariate-balanced control group. Covariates included in the model were those significantly different between the 2 groups (age, arterial hypertension, diabetes mellitus, coronary artery disease, heart failure, previous stroke). A propensity score–matched analysis (1:2) was performed due to differences in baseline characteristics between OAT groups and No-OAT. We performed nearest neighborhood matching with Mahalanobis distance (0.25-SD distance tolerance caliper). Bias reduction was assessed by comparing the standardized difference for propensity score and the other covariates before and after matching between the 2 groups (a value <10% after matching indicates inconsequential imbalance).

Univariable logistic regression analyses in unmatched and matched cohorts were performed to evaluate the association of OAT with the risk of ARDS, either at admission or during hospitalization, and presented as odds ratio (OR) with by their 95% confidence intervals (CI).

The risk of in-hospital death in unmatched and matched cohorts was calculated using univariable Cox proportional hazard regression models and presented hazard ratios (HR) with 95% confidence intervals.

Also, to identify the baseline variables associated with in-hospital mortality, we performed a multivariable Cox proportional hazard regression analysis. We used a parsimonious approach including variables with *P*<0.10 by the univariable test as a candidate for the multivariable analysis. Multicollinearity was assessed using collinearity diagnostics. The variance inflation factors showed no significant collinearity (<2.5) among the covariates.

Kaplan–Meier analyses for the assessment of survival free from in-hospital mortality in OAT vs. No-OAT groups were performed either in the whole population or in the matched cohorts, and comparisons were performed by using the log-rank test. For all test, a *P* value <0.05 was considered statistically significant. Analysis was performed by using R version 3.5.1 (R Foundation for Statistical Computing, Vienna, Austria) and SPSS version 21.0 (SPSS Inc.).

## Results

### Study Population

The clinical characteristics of the study population are reported in Table 1. The mean age was 67 ± 14 years; 174 (37%) were females. Overall, 87 patients (19%) were on OAT before admission, 54 (13%) with non-vitamin k oral anticoagulant (NOACs), and 33 (8%) with vitamin-K antagonists (VKAs). Among patients on OAT with NOACs, 14 (26%) patients received Edoxaban, 11 (20%) patients in Dabigatran, 15 (28%) in Rivaroxaban, 14 (26%) patients in Apixaban, while all VKAs patients were in warfarin. The indication for OAT was atrial fibrillation (AF) in 49 patients (56%), prosthetic heart valve in 18 patients (21%) and venous thromboembolism (VTE) in 20 patients (23%). OAT patients were older than No-OAT group (73 ± 12 vs 65 ± 15; *P*<0.001) and had a higher prevalence of hypertension (74% vs 59%; *P=0.025*), diabetes (34% vs 24%; *P*=0.057), heart failure (HF: 16% vs 6%; *P*=0.002), and history of stroke (16% vs 7%; *P*=0.007). Among OAT patients, NOACs were more frequently prescribed in patients with AF and VTE, while VKAs were reported by all patients with prosthetic mechanical heart valve (Table 2). Patients on VKAs therapy, showed more frequently coronary artery disease (CAD, 41% vs 10%; *P*=0.001) compared to those on NOAC therapy (Table 2). Considering the target international normalized ration (INR) recommended for patients on treatment with VKAs, only 4 patients resulted out of target at admission [24, 25].

**Table 1:** Baseline characteristics of study population between anticoagulated (OAT) and non-anticoagulated (No-OAT) patients

Variables	Overall population (*N* = 467)	OAT group (*N* = 87)	No-OAT group (*N* = 380)	*P*-value	Matched No-OAT group (*N* = 174)	*P*-value*
Age, years	67±14	73±12	65±14	<.001	72.5±12	.418
Female	174 (37)	33 (38)	140 (36)	.726	54 (31)	.265
Smokers	79 (17)	16 (18)	63 (17)	.824	35 (20)	.740
Dyslipidemia	119 (26)	26 (30)	93 (24)	.245	53 (31)	.924
CKD	62 (13)	15 (17)	47(12)	.212	31 (18)	.908
COPD	90 (19)	21 (24)	69 (18)	.190	43 (25)	.918
Hypertension	289 (62)	64(74)	225 (59)	.025	129 (74)	.981
Diabetes	123 (26)	30 (34)	93 (24)	.057	55 (32)	.640
CAD	71 (15)	19 (22)	52 (14)	.066	40 (23)	.834
HF	35 (8)	14 (16)	21 (6)	.002	18 (10)	.182
History of stroke	42 (9)	14 (16)	28(7)	.007	25 (14)	.583
COVID-19-related therapies						
Antiviral	265 (57)	45 (52)	220 (58)	.511	97 (56)	.538
Hydroxychloroquine	402 (86)	76 (88)	326 (86)	.643	151 (87)	.896
Antibiotics	368 (79)	64 (74)	304 (80)	.455	135 (78)	.471
Glucocorticoids	198 (42)	35 (40)	163 (43)	.847	76 (44)	.595

*CAD*, coronary artery disease; *CKD*, chronic kidney disease; *COPD*, chronic obstructive pulmonary disease; *HF*, heart failure; *No-OAT*, no oral anticoagulation therapy; *OAT*, oral anticoagulation therapy; *VTE*, venous thromboembolism

*AC group vs matched No AC group

**Table 2:** Baseline characteristics and outcome of population divided by anticoagulant type

Variables	NOAC (*N* = 54)	VKA (*N* = 33)	*P*-value
Age	73±13	74±9	.657
Female	26 (48)	9 (28)	.063
Smokers	11 (20)	6 (19)	.802
Dyslipidaemia	17 (32)	10 (31)	.908
CKD	7 (14)	7 (21)	.309
COPD	13 (24)	8 (25)	.758
Hypertension	38 (72)	25 (78)	.297
Diabetes	18 (34)	13 (41)	.566
CAD	5 (10)	13 (41)	< .001
HF	6 (12)	7 (22)	.199
History of stroke	10 (18)	5 (16)	.686
History of AF	37 (70)	14 (44)	.019
History of VTE	16 (30)	-	-
Prosthetic heart valve	-	18 (56)	-
Any ARDS	17 (32)	16 (50)	.112
ARDS at admission	12 (22)	13 (40)	.0859
ARDS during hospitalization	5 (10)	3 (9)	.978
In-hospital mortality	13 (24)	10 (31)	.361

*AF*, atrial fibrillation; *CAD*, coronary artery disease; *CKD*, chronic kidney disease; *COPD*, chronic obstructive pulmonary disease; *HF*, heart failure; *NOAC*, non-vitamin K oral anticoagulants; *VKA*, vitamin K antagonists; *VTE*, venous thromboembolism; *ARDS, acute respiratory distress syndrome*

### Chronic Anticoagulation and ARDS

In the entire population, ARDS was diagnosed in 169 patients (36%) at admission and additional 45 patients (10%) developed ARDS during the hospitalization. The rate of ARDS at admission (26% vs 28%; *P*=0.834) and ARDS developed during hospitalization (9% vs 10%; *P=*0.915) was not statistically different between OAT vs. No-OAT group. At univariable analysis, OAT was not associated with the risk of ARDS, either at admission (OR: 1.06, 95% CI 0.62–1.8) or developed during hospitalization (OR: 0.95, 95% CI 0.42–2.17).

After propensity score matching, 261 patients with balanced baseline characteristics were identified. The main baseline characteristics of the matched population are summarized in Table 1. In the matched cohorts, the rate of ARDS at admission (28% vs 27%; *P*=0.921) and ARDS developed during hospitalization (9% vs 12%; *P*=0.486) was not statistically different between OAT vs. No-OAT group. At univariable analysis, OAT was not associated with the risk of ARDS, either at admission (OR: 1.08, 95% CI 0.58–1.65) or developed during hospitalization (OR: 0.80, 95% CI 0.41–2.35). Moreover, there was no difference in the rate of ARDS according to the type of anticoagulant used (Table 2).

### Chronic Anticoagulation and Mortality

**Fig. 1:**
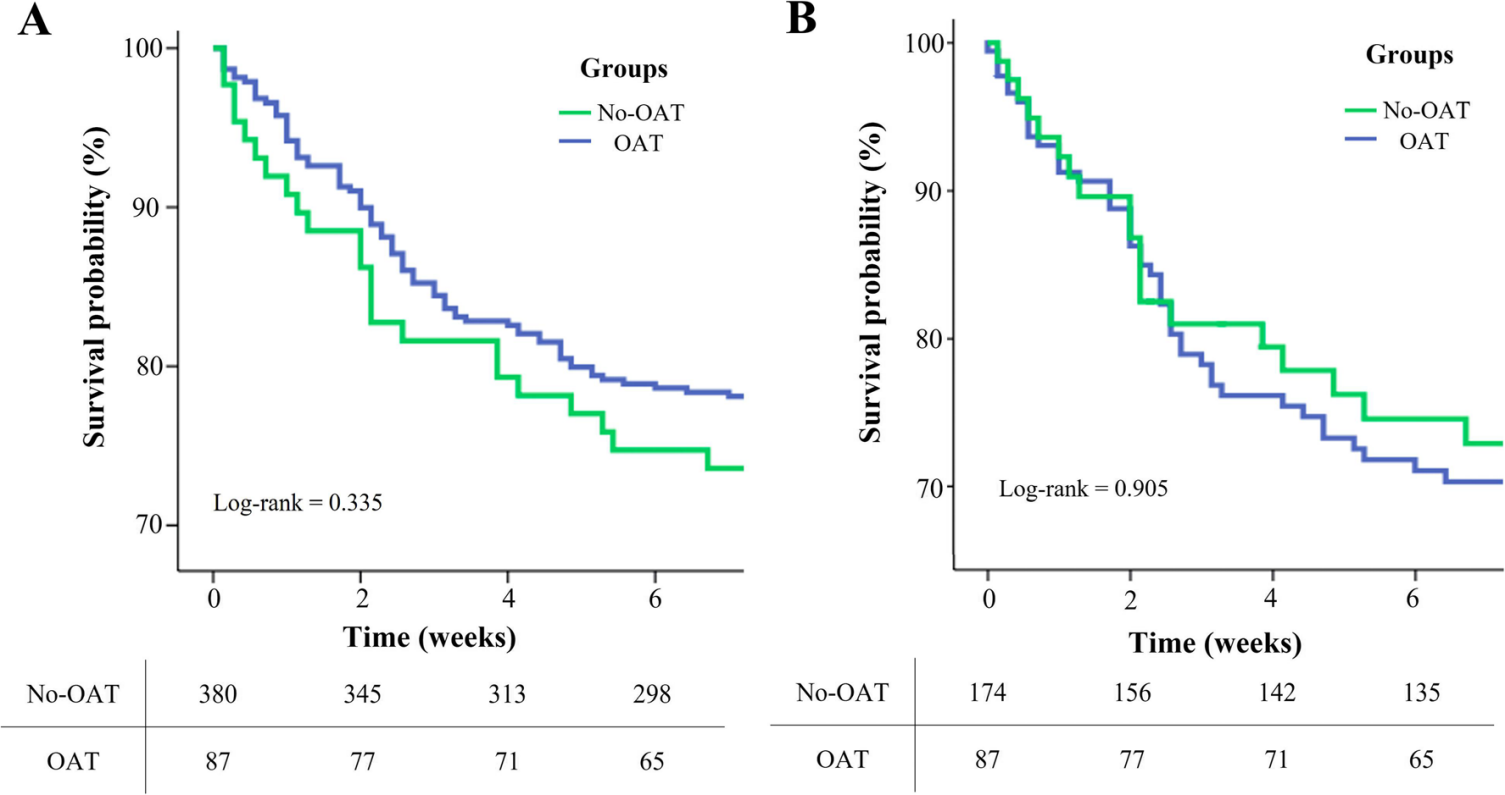
Kaplan-Meier survival free from in-hospital mortality according to anticoagulation therapy (OAT) in unmatched (panel **A**) and matched (panel **B**) cohorts

The median follow-up length was 19 days (IQR 5–27). The rate of in-hospital death was 23% in the overall population, and was not statistically different in OAT as compared to No-OAT group (22% vs 26%, *P*= 0.335); moreover, at Cox regression the oral anticoagulant use was not associated with the risk of mortality (HR: 1.26, 95% CI 0.74–2.1). Kaplan-Meier curves showed a comparable survival free from all-cause mortality between groups (log-rank = 0.335; Fig. 1). At multivariable Cox regression only male gender (HR = 1.79; 95% CI 1.14–2.78) resulted independently associated with mortality (Table 3).

**Table 3:** Cox analysis for mortality between patients with or without oral anticoagulation therapy

Variables	HR	95% CI	*P*-value
Age	1.01	0.99–1.03	.157
Male gender	1.78	1.14–2.78	.012
CAD	1.39	0.86–2.24	.174
HF	1.04	0.54–2.00	.908
CKD	1.61	0.97–2.65	.067
Hypertension	0.99	0.64–1.53	.961
History of stroke	1.02	0.52–1.99	.955
Oral anticoagulation	1.07	0.66–1.73	.785

*AF*, atrial fibrillation; *ARDS*, acute respiratory distress syndrome; *CAD*, coronary artery disease; *CKD*, chronic kidney disease; *CI*, confidential interval; *HF*, heart failure; *HR*, hazard odds ratio

In the matched population, the mortality rate did not result statistical different between study groups (26% vs 27%, *P*= 0.921). At Cox regression analysis, OAT was not associated to mortality risk (HR: 1.31, 95% CI 0.82–1.64). Kaplan-Meier curves showed a comparable survival in OAT than in No-OAT patients (log-rank = 0.905; Fig. 1). Eventually, there were no statistical difference in the rate of mortality according to the type of anticoagulant used (Table 2).

## Discussion

The main findings of the present study can be summarized as follows: 19% of patients admitted for COVID-19 were on treatment with oral anticoagulants; OAT stratified patients with older age and higher prevalence of cardiovascular (CV) comorbidities. OAT did not significantly affect the risk of severe adverse events including ARDS, either at admission or developed during the hospitalization, and in-hospital mortality. According to our data, the male gender was the only variable independently associated with the risk of mortality. Although the OAT prevalence among our study cohort was consistent with a recent German registry [20], which reported an oral anticoagulant use in the 11% of the overall population enrolled, we cannot exclude the risk of overestimation as a consequence of the selective inclusion of patients who underwent cardiology consultation.

Although the pathophysiology of ARDS in patients with COVID-19 is not completely understood, the interplay between inflammation and coagulation seems to have a pivotal role [26, 27]. The severe inflammatory response and disseminated intravascular coagulation together with virus-induced local inflammatory reactions may affect endothelial cell function leading to vessel wall damage and consequent microvascular thrombosis [13]. Functional implications include a progressive worsening of ventilation/perfusion imbalance and a loss of hypoxic vasoconstriction reflex, with a marked component of microvascular pulmonary thrombosis [28]. This mechanism, mainly driven by endothelial damage and microvascular thrombosis, suggests that microvascular lung vessels obstructive thrombo-inflammatory syndrome is a possible atypical ARDS form of patients with COVID-19 [28]

Based on this hypothesis, previous studies have investigated the protective role of chronic oral anticoagulation in hospitalized COVID-19 patients, with contrasting results [26, 28–35]. In a Swedish nationwide register-based cohort study including 459.402 patients with laboratory confirmed COVID-19, the oral anticoagulation therapy with NOACs did not result associated with risk of hospitalization for COVID-19, intensive care unit (ICU) stay and death [20]. Conversely, Denas et al. showed a significantly lower mortality in COVID-19 patients treated with OAT due to AF as compared to a propensity score matched cohort of non-anticoagulated patients, albeit without differences regarding the ICU admission [21]. However, both these studies did not evaluate the role of anticoagulant thromboprophylaxis administered during the hospitalization and did not explored whether OAT could influence the risk of ARDS at admission and/or during hospitalization.

In our study, we included all patients on pre-admission OAT, irrespective of the underlining causes; moreover, we evaluated the prevalence of ARDS at admission, which represents a marker of COVID-19 severity, in order to test the hypothetic protective role of OAT on the hypercoagulable state that may lead to pulmonary microthrombosis.

The in-hospital discontinuation of OAT was considered an exclusion criterion, to avoid bias deriving from the out-of-range therapeutic periods caused by in-hospital anticoagulation treatment switching or discontinuation. Also, patient treated with heparin before the hospitalization were excluded in order to avoid potential confounding derived from the use of different type of anticoagulants.

Some authors hypothesized that microvascular pulmonary thrombosis in COVID-19-induced pneumonia is sustained from a complex interplay between clotting system activation and immuno-mediated inflammatory response, two processes mutually reinforced each other [36, 37]. OAT, by targeting one single pathway, would not seem to influence significantly the progression of SARS-CoV-2 infection and, eventually, the natural history of the disease.

Pulmonary and extra-pulmonary microvascular thrombosis may considerably contribute to the acute lung injury and multiple organ dysfunction, which characterize the severe forms of COVID-19 (26). In our analysis, only male sex was found to be significantly associated to an increased in-hospital mortality risk. Our findings are consistent with those reported in previous observational studies [38] and meta-analysis [39], which demonstrate a higher mortality risk of men over women. Male gender may be associated with a worse prognosis during COVID-19 through several potential mechanisms: first, men largely show behaviors potentially harmful for health, such as smoking, poor diet, or a sedentary lifestyle, and more concomitant diseases [40]; second, hormones seem to play an important role in COVID-19 pathophysiology; estrogens may improve the immune response, as they enhance the proliferation of T lymphocytes and attenuate the cytokine storm, while testosterone could have a negative effect [41]; Third, the higher expression of angiotensin converting enzyme 2 (ACE2) receptor in the Leydig cells of men, could lead to a more diffuse alveolar damage than in women [42]

Furthermore, also the anticoagulation type (NOACs vs. VKAs) did not seem to significantly impact on the disease severity and prognosis of hospitalized COVID-19 patients.

## Study Limitations

Our study is limited by the retrospective design, the relatively small simple size of patients on anticoagulation therapy. Compared with previous report, we found a high rate of ARDS and mortality, which may reflect the high-risk profile of our study population [43]. Thus, the role of OAT as an effect modifier on the risk of ARDS and/or death cannot be generalized to lower-risk COVID-19 populations. Second, the use of VKAs or NOACs was considered on the class effect basis, since the limited power of our study would not allow sub-analysis addressing to the use of specific OAT agents.

Third, although we collected the INR values at admission for patients on treatment with VKAs, and we reported how many of them were out of target at admission, data on laboratory coagulation parameter during the hospitalization were not available in this register [40].

Fourth, since the register included only hospitalized patients with COVID-19, our results cannot be generalized to the overall population of SARS-CoV-2 infected patients, who are asymptomatic or poorly symptomatic in most of the cases; moreover, we cannot determine if OAT prevented any SARS-CoV-2 infected patients from developing severe symptoms. Larger prospective studies are needed to confirm our preliminary findings.

## Conclusions

In this multicenter registry enrolling consecutive hospitalized patients with COVID-19, there was no significant association between OAT, either with NOACs or VKAs, and the severity of the disease in terms of ARDS, at admission or developed during hospitalization, and of in-hospital mortality.

## Data Availability

Not applicable
